# Pathogenicity and escape to pre-existing immunity of a new genotype of swine influenza H1N2 virus that emerged in France in 2020

**DOI:** 10.1186/s13567-024-01319-5

**Published:** 2024-05-21

**Authors:** Céline Deblanc, Stéphane Quéguiner, Stéphane Gorin, Gautier Richard, Angélique Moro, Nicolas Barbier, Gérald Le Diguerher, Frédéric Paboeuf, Séverine Hervé, Gaëlle Simon

**Affiliations:** 1 Swine Virology Immunology Unit, Ploufragan-Plouzané-Niort Laboratory, French Agency for Food, Environmental and Occupational Health and Safety (ANSES), 22440 Ploufragan, France; 2 SPF Pig Production and Experimentation, Ploufragan-Plouzané-Niort Laboratory, French Agency for Food, Environmental and Occupational Health and Safety (ANSES), 22440 Ploufragan, France

**Keywords:** Influenza virus, swine, H1N2 subtype, reassortant virus, vaccination, post-infection immunity

## Abstract

In 2020, a new genotype of swine H1N2 influenza virus (H1_av_N2–HA 1C.2.4) was identified in France. It rapidly spread within the pig population and supplanted the previously predominant H1_av_N1-HA 1C.2.1 virus. To characterize this new genotype which is genetically and antigenically distant from the other H1_av_Nx viruses detected in France, an experimental study was conducted to compare the outcomes of H1_av_N2 and H1_av_N1 infections in pigs and evaluate the protection conferred by the only inactivated vaccine currently licensed in Europe containing an HA 1C (clade 1C.2.2) antigen. Infection with H1_av_N2 induced stronger clinical signs and earlier shedding than H1_av_N1. The neutralizing antibodies produced following H1_av_N2 infection were unable to neutralize H1_av_N1, and vice versa, whereas the cellular-mediated immunity cross-reacted. Vaccination slightly altered the impact of H1_av_N2 infection at the clinical level, but did not prevent shedding of infectious virus particles. It induced a cellular-mediated immune response towards H1_av_N2, but did not produce neutralizing antibodies against this virus. As in vaccinated animals, animals previously infected by H1_av_N1 developed a cross-reacting cellular immune response but no neutralizing antibodies against H1_av_N2. However, H1_av_N1 pre-infection induced a better protection against the H1_av_N2 infection than vaccination, probably due to higher levels of non-neutralizing antibodies and a mucosal immunity. Altogether, these results showed that the new H1_av_N2 genotype induced a severe respiratory infection and that the actual vaccine was less effective against this H1_av_N2-HA 1C.2.4 than against H1_av_N1-HA 1C.2.1, which may have contributed to the H1_av_N2 epizootic and dissemination in pig farms in France.

**Supplementary Information:**

The online version contains supplementary material available at 10.1186/s13567-024-01319-5.

## Introduction

Swine influenza is a widespread respiratory disease in pig herds characterized by dyspnea, nasal discharge, cough, fever, lethargy and loss of appetite for a period of 5–7 days [1]. The disease is caused by swine influenza A viruses (swIAV), which belong to the *Orthomyxoviridae* family. Three swIAV subtypes, characterized by the two surface glycoproteins, the hemagglutinin (HA) and the neuraminidase (NA), are circulating in the pig population, i.e., H1N1, H1N2 and H3N2. However, different genetic lineages are distinguished within each subtype, depending on the origins of the HA and NA genes. In the early 2010s, four lineages were mostly detected in Europe: the “avian-like swine H1N1” (H1_av_N1), the “human-like reassortant swine H3N2” (H3N2), the “human-like reassortant swine H1N2” (H1_hu_N2) and the “pandemic-like swine H1N1” lineages [2]. However, in an intensive breeding context promoting persistent forms of influenza as well as co-circulations of several swIAV lineages, reassortant viruses combining gene segments from these main swIAVs, or even genes from seasonal human viruses, were more and more frequently detected, increasing the diversity of swIAV genotypes in Europe [3]. The relative proportions of the different lineages and genotypes identified in the various European countries has continued to evolve giving each country its own specific panel of circulating lineages [3–9]. That said, the H1_av_N1 (HA clade 1C.2.1 and other genes from “Eurasian-avian like swine” (EA) lineage) virus was still predominant in France at the end of the 2010s, a situation that has been unchanged for 40 years [6]. However, in 2020, the swIAV surveillance national network highlighted an important change in the distribution of swIAV lineages, with a rapid and wide dissemination of an H1_av_N2 lineage [10]. Thus, H1_av_N2 virus strains accounted for 64% of swIAV strains detected and identified in 2020, whereas this lineage was only sporadically detected before [10, 11]. It was responsible for a marked epizootic, became established in the pig population and remained the most frequently identified in France in 2021 and 2022 [11, 12].

The H1_av_N2 virus that emerged in 2020 in Brittany, in the Western part of France and spread on the territory, contained an HA gene from clade 1C.2.4 [13], a NA gene from the “H3N2 Gent-like” lineage and internal genes from the EA lineage [10]. Phylogenetic analyses indicated that any of the eight genomic segments originated from swIAV that circulated in France previously, but from a H1_av_N2 swIAV that had become enzootic in Denmark since 2003 following a H1_av_N1 × H3N2 reassortment [14]. Then, this Danish-origin H1_av_N2 virus progressively spread in Germany [15], Italy [4] and Spain [7]. It was detected on two occasions in South-Western France in 2015, but did not settle in the pig population at that time. Genetic analysis of these two 1C.2.4 strains from 2015 revealed a number of differences compared to other 1C.2 viruses detected in France, including an amino acid deletion at position 146 in HA [6]. Interestingly, the H1_av_N2 virus that was newly introduced in 2020 showed additional genetic differences in comparison to other Danish-origin H1_av_N2 strains circulating in Europe and previously identified in France, including a double deletion at positions 146–147 and non-synonymous modifications in the HA, fixed in the receptor binding site and antigenic sites [16]. Such modifications might have induced changes in cell receptor affinity, viral multiplication, pathogenicity and/or antigenic properties, changes that could have played a role in the ability of this virus to spread so rapidly through the pig population regardless of pre-existing immunity, whether post-infectious or post-vaccinal.

This study aimed to characterize the phenotype of this new H1_av_N2 genotype that has become enzootic in France in terms of antigenicity and pathogenicity. Especially, we compared the outcomes of H1_av_N2 (HA clade 1C.2.4) infection to that of H1_av_N1 (HA clade 1C.2.1) in experimentally-inoculated pigs, as well as the protection conferred by the only vaccine currently licensed in Europe that contains an HA-1C antigen. Moreover, we evaluated the impact of pre-existing immunity induced by H1_av_N2 or H1_av_N1 primo-infections in unvaccinated or vaccinated pigs towards a second infection with the H1_av_N2 emerging genotype.

## Materials and methods

### Ethics statement

The experiment was performed in the facilities of the French Agency for Food, Environmental, and Occupational Health and Safety (ANSES, Ploufragan, France) which has an agreement for animal experimentation (ANSES registration number D227451). The animal experiment protocol was approved by the French National Committee for Ethics in Animal Experimentation ANSES/ENVA/UPEC n°16 and authorized by the French Ministry for Research (approval No. APAFIS#33432–2021100717445083 v2).

### Vaccine and virus strains

The vaccine used in this study was the Respiporc^®^ Flu3 vaccine (CEVA, Libourne, France), a commercial trivalent inactivated vaccine containing antigens representative of the “avian-like swine H1N1” (H1_av_N1—HA-1C.2.2), the “human-like reassortant swine H3N2” (H3N2), and the “human-like reassortant swine H1N2” (H1_hu_N2) lineages [17], that were the three most widespread enzootic swIAV lineages circulating in Europe in the 2000s.

The challenge strains A/Swine/France/29-200272-01/2020 (272/20-H1_av_N1-HA-1C.2.1) and A/Swine/France/35-200154-01/2020 (154/20-H1_av_N2—HA-1C.2.4) were selected as genotype-specific representative strains among collections of the French National Reference Laboratory for Swine Influenza (ANSES, Ploufragan, France). They were both isolated in 2020 from nasal swabs taken from pigs with acute respiratory disease thanks to swIAV passive surveillance, then propagated and titrated in Madin–Darby canine kidney (MDCK) cells following a standard procedure [18].

As NA, especially of N2 subtype, may induce some reaction in hemagglutination inhibition (HI) tests [19], a third strain, i.e. A/Swine/Cotes d’Armor/0186/2010 (186/10-H1_av_N2—HA 1C.2.1), was used as another antigen in cross- HI tests and virus neutralization (VN) tests in addition to both challenge strains. This strain was representative of the main H1_av_N2 genotype detected in France before 2020, with an HA gene from clade 1C.2.1, a NA gene from the “H1N2 Scotland/94-like” lineage and internal genes from the EA lineage.

### Genetic distance analyses

HA amino acid (aa) sequences of swIAV strains used in this study and vaccine strains were deduced from nucleotide sequences (accession numbers are given in Table 2) and degrees of homology between sequences were assessed by calculating percent identity after alignments using the webserver CLUSTAL Omega [20].

### Experimental design and sample collection

Thirty-six specific pathogen-free (SPF) pigs were obtained from the ANSES pig herd. At 4 weeks of age, animals were randomly allocated into six groups and housed in separate air-filtrated biosecurity level 3 units. After one week of acclimation period, half of the groups were prime-boost vaccinated with Respiporc^®^ Flu3 vaccine at 3 week interval, i.e. they received an intra-muscular injection (2 mL per dose) at 5 and 8 weeks of age, respectively (Table 1). At 9 weeks of age [day 0 (D0)], one unvaccinated group and one vaccinated group were inoculated intra-tracheally with 10^6 ^TCID_50_ (50% tissue culture infectious dose), in a volume of 5 mL, of the 272/20-H1_av_N1 strain (H1N1 and H1N1 VACC groups, respectively), or the 154/20-H1_av_N2 strain (H1N2 and H1N2 VACC groups, respectively). The two last groups received 5 mL of Eagle’s Minimum Essential Medium and were used as controls (CONTROL and CONTROL VACC groups). Three weeks later, at 12 weeks of age (D21), all swIAV-inoculated animals were re-inoculated with the 154/20-H1_av_N2 virus while the study stopped for the two control groups for logistical reasons. Re-inoculated animals were followed for a further 4 week period, i.e. until 16 weeks of age (D49).

**Table 1:** **Experimental design**

Group ID	Vaccination with Respiporc^®^ Flu3 at 5 (D-28) and 8 (D-7) weeks of age	Inoculum	
		1^st^ challenge at 9 weeks of age (D0)	2^nd^ challenge at 12 weeks of age (D21)
H1N1	No	272/20-H1_av_N1	154/20-H1_av_N2
H1N2	No	154/20-H1_av_N2	154/20-H1_av_N2
CONTROL	No	EMEM	none
H1N1 VACC	Yes	272/20-H1_av_N1	154/20-H1_av_N2
H1N2 VACC	Yes	154/20-H1_av_N2	154/20-H1_av_N2
CONTROL VACC	Yes	EMEM	none

272/20-H1_av_N1 = A/Swine/France/29-200272-01/2020 (H1_av_N1-HA clade 1C.2.1), 154/20-H1_av_N2 = A/Swine/France/35-200154-01/2020 (H1_av_N2-HA clade 1C.2.4), EMEM = Eagle’s Minimum Essential Medium (mock-inoculation).

Every day throughout the study, clinical signs were recorded individually, while coughs and sneezes were counted for 15 min in each room. Behavior and respiratory signs registered for each pig were scored as followed: 0 = no clinical signs, 1 = decreased liveliness, 2 = rapid breathing, 3 = rapid breathing + decreased liveliness. Animals were weighed daily during the week following the inoculations, otherwise once a week.

For two weeks following the challenges, individual oral fluids were daily collected using a fragment of wipe (SODIBOX, Nevez, France) and nasal swabs were taken with Virocult^®^ (MWE medical wire, Corsham, UK) every two or three days, for virus excretion measurements. Additionally, rectal swabs were taken on the same days as nasal swabs in unvaccinated groups.

At D-28 and then once a week from D0 until the end of the study, blood samples were taken with or without heparin, in order to collect peripheral blood mononuclear cells (PBMC) or serum, respectively, for monitoring immune responses. Additional sera were collected at D1 and D3 for measurements of inflammatory and/or antiviral responses. All samples were stored at −20 °C or −70 °C until use, except PBMC which were analyzed extemporaneously.

All animals were euthanised and necropsied at the end of the experiment, between D49 and D51. Lungs were removed in toto and macroscopic lesions were estimated visually by assigning one score out of 28, as previously described [21]. If a pneumonia lesion was observed, a fragment of lung was collected to test for the presence of the virus.

### Virus genome quantification and virus titration

The presence or absence of the virus in the different collected samples was assessed by in-house swIAV M gene RT-qPCR and, when possible, the swIAV M gene was quantified. For swIAV detection in oral fluids, and in nasal and rectal swab supernatants, total RNA was extracted from 150 µL of sample using the ID Gene^™^ Mag Fast 384 Extraction Kit (Innovative Diagnostics, Grabels, France) on the KingFisher^™^ Flex Purification System (ThermoFisher Scientific, Waltham, MA, USA). Then, RNA was tested by duplex M/β-actin RT-qPCR for the detection of the swIAV M gene, as previously described [22]. For normalization, viral RNA amounts quantified in nasal swab supernatants were expressed as the M gene copy number per 10^6^ copies of β-actin gene. Area under the curves (AUC) were calculated using GraphPad Prism version 9.5.0 for Windows (GraphPad Software, San Diego, CA, USA) to evaluate the total shedding of viral RNA in each group during the study. The presence of the swIAV genome in lungs was tested using the same PCR method, without quantification.

Infectious swIAV particles secreted in nasal swab supernatants obtained at D3 and D5 from pigs in H1N2 and H1N2 VACC groups were titrated via cytopathic effect assay on MDCK cells as previously described [23]. Virus titers were calculated using the formula by Reed and Muench and expressed as TCID_50_/mL. Given the fact that nasal secretion bulk might differ from sample to sample, titers were additionally normalized per 10^6^ copies of β-actin.

### Cytokine measurements

Porcine interleukine (IL)-6 was measured in serum using an ELISA commercial kit (Bio-Techne, Minneapolis, MN, USA) according to the manufacturer’s instructions. Interferon (IFN)-α was measured using an in-house ELISA [24].

### Hemagglutination inhibition assay

Hemagglutination inhibition (HI) tests implemented for evaluation of antigenic distances between swIAV strains were performed according to standard protocols [18]. These tests were carried out using a panel of seven hyperimmune swine sera previously produced in SPF pigs following inoculation of A/Swine/Côtes d’Armor/0388/09 (H1_av_N1, HA clade 1C.2.1); A/Swine/Côtes d’Armor/0186/2010 (H1_av_N2, HA clade 1C.2.1); A/Swine/France/35–200154/2020 (H1_av_N2, HA clade 1C.2.4); A/Swine/France/65–150242/15 (H1_av_N2, HA clade 1C.2.4); A/Swine/Sarthe/0255/10 (H1N1pdm, HA clade 1A.3.3.2), A/Swine/Scotland/410440/94 (H1_hu_N2, HA clade 1B.1) and A/Swine/Flandres/1/98 (H3N2), respectively [6]. A serum obtained from one unvaccinated and uninfected SPF pig was also included in the panel as a negative control, as well as a serum from a vaccinated sow as a positive control for detection of post-vaccination antibodies. This sow received five injections of Respiporc^®^ Flu3 vaccine and the serum was collected four weeks after the last boost [22]. HI tests were performed with a starting serum dilution of 1:10 and with 0.5% chicken red blood cells. Strains 272/20-H1_av_N1 (HA clade 1C.2.1), 186/10-H1_av_N2 (HA clade 1C.2.1) and 154/20-H1_av_N2 (HA clade 1C.2.4) were used at a concentration of four hemagglutinating units (HAU)/well. HI titers were expressed as the reciprocal of the highest dilution of serum inhibiting four HAU. A serum was considered positive when HI titer ≥ 20.

Anti-HA antibodies were quantified in sera from pigs included in the experimental study by a similar HI test. HI titers were log_2_ transformed for statistical analysis and reported as means of HI titers per group.

### Virus neutralization assay

Virus neutralization (VN) tests implemented to assess antigenic distances between swIAV strains were performed as previously described [25], using the same panel of hyperimmune swine sera as for HI tests. The neutralizing antibody titer was determined as the reciprocal of the highest dilution of serum that prevents virus infection of MDCK determined by the absence of cytopathic effect in half of the duplicate wells. A serum was considered positive when VN titer ≥ 20.

Neutralizing antibodies targeting either the 272/20-H1_av_N1 strain and/or the 154/20-H1_av_N2 strain were quantified in sera collected throughout the experimental study by a similar VN assay, with a starting serum dilution of 1:20. The titers were log_2_ transformed in order to calculate the mean VN titer in each group of pigs.

### Anti-swIAV IgG and IgA detection

The immunoglobulins G (IgG) directed against the viral nucleoprotein (NP) were quantified in sera with the ID Screen^®^ Influenza A Nucleoprotein Swine Indirect kit (Innovative Diagnostics, Grabels, France) following the manufacturer’s instructions (dilution 1:100), and in nasal swabs using an adapted protocol (dilution 1:2). Anti-NP immunoglobulins A (IgA) were detected in nasal swabs (dilution 1:2) with the same kit as IgG, with a modified protocol using in-house controls and a goat anti‐pig IgA antibody HRP conjugate (A100-102P, Bethyl—Fortis Life Sciences, Montgomery, TX, USA) at a 1:3000 dilution as a conjugated antibody. Antibody levels were expressed in sample‐to‐positive (S/P) ratios.

### ELISPOT IFN-γ

PBMC were isolated by Ficoll density gradient centrifugation using LeucoSep tubes (Greiner Bio One, Les Ulis, France). SwIAV specific IFN-γ secreting cells (IFNγ-SC) were quantified in triplicate by enzyme-linked immunospot (ELISPOT) as previously described [25], using a 18 h stimulation of 4 × 10^5^ PBMC with either the 272/20-H1_av_N1 strain or the 154/20-H1_av_N2 strain, at a multiplicity of infection of 0.5. The number of spots per well was counted using an ImmunoSpot S6 UV Analyser (CT, Shaker Heights, OH, USA). The number of IFNγ-SC was calculated by subtracting the number of non-specific spots that were obtained for the negative stimulation (cell culture medium) from the number of spots obtained for the viral stimulation, then expressed per million of PBMC.

### Statistical analyses

For all data, experimental groups were compared applying the non-parametric Kruskal–Wallis test. When significant differences were obtained with this test, a BH-corrected Wilcoxon test was then performed for pairwise comparisons. To study the evolution of a group over time, this test was applied for paired samples. Analyses were performed using R software (version 4.1.2) and significant differences were considered when *p* < 0.05.

## Results

### Genetic and antigenic distances between H1_av_ strains

Homology between HA sequences of strains from clade 1C.2.1, 1C.2.4 and of the vaccine antigen are reported in Table 2. The HA 1C.2.4 from the 154/20-H1_av_N2 strain showed a homology of 90.23% and 87.57% with the HA 1C.2.1 from the 186/10-H1_av_N2 strain, representative of the main H1_av_N2 genotype detected in France before the emergence of the new H1_av_N2 genotype represented by the 154/20-H1_av_N2 virus, and from the 272/20-H1_av_N1 strain, respectively. The 154/20-H1_av_N2 strain showed a more important divergence with the HA 1C.2.2 antigen contained in the vaccine than the 272/20-H1_av_N1 strain, since the percent identity reached 90.05% for the 154/20-H1_av_N2 strain versus 91.87% for the 272/20-H1_av_N1 strain.

**Table 2:** **Homology between H1**_**av**_** virus strains**

Strain name (lineage)	Used as	H1 clade	GenBank accession number of the HA protein	aa identity (%) with challenge strains	
				A/Swine/France/29-200272-01/2020 (H1_av_N1 – HA 1C.2.1)	A/Swine/France/35-200154-01/2020 (H1_av_N2 – HA 1C.2.4)
A/Sw/France/29–200272-01/2020 (H1_av_N1)	Challenge strain	1C.2.1	QUQ74233	100	87.57
A/Sw/Côtes d’Armor/0186/2010 (H1_av_N2)	Reference antigen	1C.2.1	ATU89338	93.29	90.23
A/Sw/France/35–200154-01/2020 (H1_av_N2)	Challenge strain	1C.2.4	QUQ74329	87.57	100
A/Sw/Haseluenne/IDT2617/2003 (H_av_N1)	Vaccine antigen^a^	1C.2.2	ACR39183	91.87	90.05

^a^Included in Respiporc^®^ Flu3.

Antigenic distances between strains 272/20-H1_av_N1 (HA clade 1C.2.1), 186/10-H1_av_N2 (HA clade 1C.2.1) and 154/20-H1_av_N2 (HA clade 1C.2.4) were evaluated in cross-HI and cross-VN tests (Tables 3 and 4, respectively).

**Table 3:** **Evaluation of antigenic distances between H1**_**av**_** strains by cross-HI tests**

	Hyperimmune serum containing antibodies directed against							Post-vaccination (Respiporc^®^ Flu3) serum
Virus strain (lineage—HA clade)	A/Sw/Cotes d’Armor/0388/09 H1_av_N1—clade 1C.2.1	A/Sw/Cotes d'Armor/0186/2010 H1_av_N2—clade 1C.2.1	A/Sw/France/35-200154/2020 H1_av_N2—clade 1C.2.4	A/Sw/France/65–150242/2015 H1_av_N2—clade 1C.2.4	A/Sw/Sarthe/0255/10 H1N1pdm—clade 1A.3.3.2	A/Sw/Scotland/410440/94 H1_hu_N2—clade 1B.1	A/Sw/Flandres/1/98 H3N2	
A/Sw/France/29–200272-01/2020 (H1_av_N1—clade 1C.2.1)	1280	640	10	160	40	10	< 10	160
A/Sw/Côtes d’Armor/0186/2010 (H1_av_N2—clade 1C.2.1)	640	**640**	40	80	80	10	< 10	160
A/Sw/France/35–200154-1/2020 (H1_av_N2—clade 1C.2.4)	20	10	**1280**	160	< 10	< 10	20	10

Hemagglutination inhibition (HI) titers of a panel of hyperimmune and post-vaccination sera towards the H1_av_N1-HA clade 1C.2.1 and H1_av_N2-HA clade 1C.2.4 challenge strains (representing the main genotypes circulating in France in 2020), and a H1_av_N2-HA 1C.2.1 strain (representing the H1_av_N2 genotype mainly detected in France before 2020). A serum sample was positive towards a given antigen when HI titer ≥ 20. Results in bold report strictly homologous reactions.

**Table 4:** **Evaluation of antigenic distances between H1**_**av**_** strains by cross-VN tests**

	Hyperimmune serum containing antibodies directed against							Post-vaccination (Respiporc^®^ Flu3) serum
Virus strain (lineage – HA clade)	A/Sw/Cotes d'Armor/0388/09 H1_av_N1—clade 1C.2.1	A/Sw/Cotes d’Armor/0186/2010 H1_av_N2—clade 1C.2.1	A/Sw/France/35–200154/2020 H1_av_N2—clade 1C.2.4	A/Sw/France/65–150242/2015 H1_av_N2—clade 1C.2.4	A/Sw/Sarthe/0255/10 H1N1pdm—clade 1A.3.3.2	A/Sw/Scotland/410440/94 H1_hu_N2—clade 1B.1	A/Sw/Flandres/1/98 H3N2	
A/Sw/France/29-200272-01/2020 (H1_av_N1—clade 1C.2.1)	1920	480	< 20	80	< 20	< 20	< 20	320
A/Sw/Côtes d’Armor/0186/2010 (H1_av_N2—clade 1C.2.1)	960	**2560**	30	80	60	< 20	< 20	480
A/Sw/France/35-200154-1/2020 (H1_av_N2—clade 1C.2.4)	60	20	**7680**	160	< 20	< 20	< 20	30

Virus neutralization (VN) titers of a panel of hyperimmune sera and post-vaccination serum towards the H1_av_N1-HA clade 1C.2.1 and H1_av_N2-HA clade 1C.2.4 challenge strains representing the main strains circulating in France in 2020, and a H1_av_N2-HA 1C.2.1 strain representing the H1_av_N2 genotype mainly detected in France before 2020. A serum sample was positive towards a given antigen when VN titer ≥ 20. Results in bold report homologous reactions.

In HI tests, no reaction was observed with the negative serum, whatever the strain tested (HI titer < 10). The 272/20-H1_av_N1 challenge strain reacted very well with sera containing anti-HA clade 1C.2.1 antibodies, with HI titers reaching 640–1280, and to a lesser extent with the post-vaccination serum (HI titer: 160) (Table 3). A reaction was also measured between this strain and antibodies (Ab) targeting a H1_av_N2 (HA clade 1C.2.4) strain sporadically detected in 2015 (HI titer: 160), but not with Ab against the new H1_av_N2 (HA clade 1C.2.4) virus detected in 2020 (HI titer: 10). Similar results were obtained with the 186/10-H1_av_N2 strain harboring an HA from clade 1C.2.1. Regarding the 154/20-H1_av_N2 (HA clade 1C.2.4) strain, a high HI titer was obtained in homologous reaction condition (HI titer: 1280), which was three times higher than with the anti-HA clade 1C.2.4—2015 serum (HI titer: 160). Very low reactions were obtained with Ab targeting viruses with HA clade 1C.2.1 (HI titers: 10–20) or with post-vaccine Ab (HI titer: 10).

Cross-VN tests (Table 4) gave outcomes similar to the cross-HI tests. Thus, the virus strains used in VN tests were not neutralized by the serum used as negative control (VN titer < 20). The 272/20-H1_av_N1 strain was neutralized by Ab targeting virus strains with HA from clade 1C.2.1 (VN titers: 1920 and 480) and by post-vaccine Ab (VN titer: 320), and to a lesser extent by Ab directed against the H1_av_N2 (HA clade 1C.2.4) strain from 2015 (VN titer: 80) (Table 4). However, the 272/20-H1_av_N1 strain was not neutralized by Ab directed against the H1_av_N2 (HA clade 1C.2.4) strain detected in 2020 (VN titer < 20). Similar results were obtained for the H1_av_N2 (HA clade 1C.2.1) strain. Conversely, while the 154/20-H1_av_N2 (HA clade 1C.2.4) strain was efficiently neutralized in homologous reaction (VN titer: 7680), both sera containing Ab directed against strains with an H1_av_ from clade 1C.2.1 reacted poorly, with VN titers seven to eight times lower (VN titers: 60 and 20, respectively). Ab contained in the serum taken from a vaccinated sow also failed to efficiently neutralize the 154/20-H1_av_N2 strain, obtaining a VN titer of 30 only, which was three times lower than that measured for the 272/20-H1_av_N1 strain. Moreover, Ab directed against the H1_av_N2 (HA clade 1C.2.4) strain from 2015 did not neutralize the 2020 H1_av_N2 (HA clade 1C.2.4) strain any better than those from H1_av_N1 anti-serum (VN titer: 160).

### Clinical signs and lesions induced by 272/20-H1_av_N1 and 154/20-H1_av_N2 challenges

Unvaccinated and vaccinated pigs were inoculated with the 272/20-H1_av_N1 strain (H1N1 group and H1N1 VACC group, respectively), or the 154/20-H1_av_N2 strain (H1N2 group and H1N2 VACC group, respectively), or mock-inoculated (CONTROL group and CONTROL VACC group, respectively) at D0, then all pre-infected pigs were infected a second time at D21 with the 154/20-H1_av_N2 strain.

No clinical signs were observed in the control groups throughout the study.

**Figure 1:**
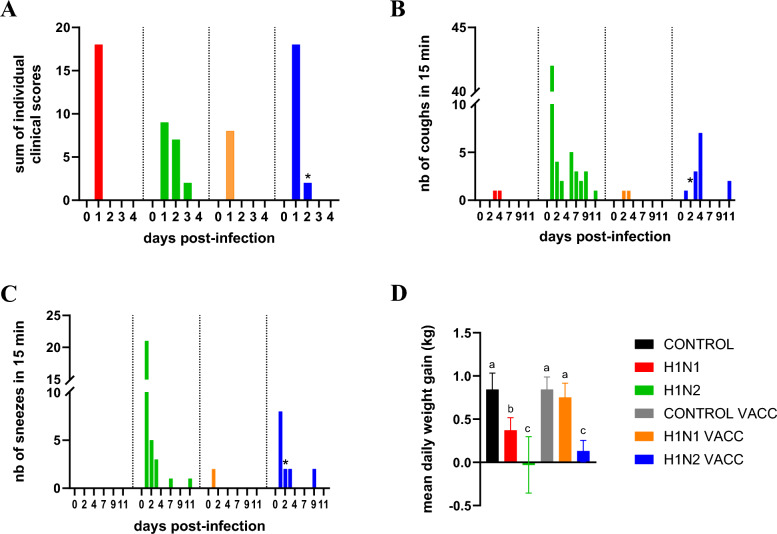
**Clinical signs collected daily from D0 to D11.** **A** Sums of individual clinical scores. Behavior and respiratory signs registered for each pig (*n* = 6 per group) were scored as followed: 0 = no clinical signs, 1 = decreased liveliness, 2 = rapid breathing, 3 = rapid breathing + decreased liveliness, then individual scores were added together for giving a total score for the group. **B** Numbers (nb) of coughs and (**C**) nb of sneezes heard in each room in 15 min. **D** Mean daily weight gains (± standard deviation) of pigs between D0 and D4. Significant differences between groups are indicated by different letters. * Only 5 pigs in the H1N2 VACC group from D2, instead of 6. H1N1 and H1N1 VACC: groups challenged at D0 with the 272/20-H1_av_N1 strain (HA clade 1C.2.1). H1N2 and H1N2 VACC: groups challenged at D0 with the 154/20-H1_av_N2 strain (HA clade 1C.2.4).

After the first swIAV-challenge, all pigs in the H1N1 group showed rapid breathing and a decreased liveliness at D1 but recovered the next day. In the H1N2 group, these clinical signs were prolonged as all animals, except one, presented increased breathing and/or decreased liveliness from D1 to D3 (Figure 1A). In addition, infection with 154/20-H1_av_N2 induced more coughing and sneezing than 272/20-H1_av_N1, as 42 coughs and 21 sneezes were heard in 15 min in the H1N2 room at D1 whereas none were heard in the H1N1 room (Figures 1B, C). The number of coughs and sneezes decreased from D2 but they persisted for about ten days. In both swIAV inoculated groups, the infection induced a decrease in the mean daily weight gain (MDWG) calculated over the D0/D4 period but this reduction was significantly more important in the H1N2 group than in the H1N1 group (Figure 1D). Indeed, the MDWG was—0.029 ± 0.327 kg in the H1N2 group, against 0.371 ± 0.146 kg in the H1N1 group and 0.842 ± 0.190 kg in the CONTROL group.

In the H1N1 VACC group, all animals had a normal liveliness and only 4/6 pigs presented a rapid breathing at D1, indicating that vaccination reduced the clinical signs induced by the 272/20-H1_av_N1-infection in naïve pigs (Figure 1A). In addition, the MDWG in the H1N1 VACC group was similar to that in the CONTROL VACC group, i.e. 0.750 ± 0.167 kg and 0.842 ± 0.146 kg, respectively (Figure 1D). Such a reduction of clinical signs was not observed in the H1N2 VACC group as compared to the H1N2 group, as all vaccinated animals were still severely affected at D1, showing rapid breathing and decreased vitality even if the numbers of coughing and sneezing were slightly reduced (Figures 1A–C). To be note that one of the animals from H1N2 VACC group had to be euthanized at D1 for ethical reasons because it did not respond to any stimuli and its body temperature began to fall. At the necropsy, this pig showed macroscopic lung lesions reaching the score of 13/28. Pneumonia lesions were observed on all pulmonary lobes, with in particular, important damages to the azygos and the right cardiac and diaphragmatic lobes, and the swIAV genome was detected in all tested lobes (right apical, cardiac and diaphragmatic lobes). Vaccination also did not prevent MDWG reduction after 154/20-H1_av_N2 infection. Indeed, the MDWG over the D0/D4 period was 0.130 ± 0.124 kg in the H1N2 VACC group, which was similar to the H1N2 group and significantly different from the H1N1 VACC and the CONTROL VACC groups (Figure 1D). Regarding the rectal temperature, five over the six H1N2 VACC pigs presented hyperthermia (rectal temperature > 40 °C) at D1 against only two pigs in the H1N1 VACC group. However, the mean rectal temperatures were 40.6 ± 0.5 °C and 39.9 ± 0.6 °C in H1N2 VACC and H1N1 VACC groups, respectively, which were not significantly different from each other (*p* = 0.108), and different from the CONTROL VACC group (39.1 ± 0.2 °C).

After the second challenge with the 154/20-H1_av_N2 strain at D21, no clinical signs and hyperthermia were observed in the four infected groups and the MDWG calculated over the D21/D25 period were similar in the four groups (0.938 ± 0.197 kg, 1.058 ± 0.164 kg, 1.050 ± 0.164 kg and 1.040 ± 0.110 kg in H1N1, H1N2, H1N1 VACC and H1N2 VACC groups, respectively). At necropsy, some pneumonia lesions were still present on the upper lobes of 4/6 pigs in the H1N2 group, while no such observation was made in the other groups. For these four H1N2 pigs, the pulmonary macroscopic lesions reached the average score of 6 (± 3.7)/28 and viral genome was detected in the lung fragments (data not shown).

### Virus shedding

No virus was detected in the control pigs at any time during the experiment.

**Figure 2:**
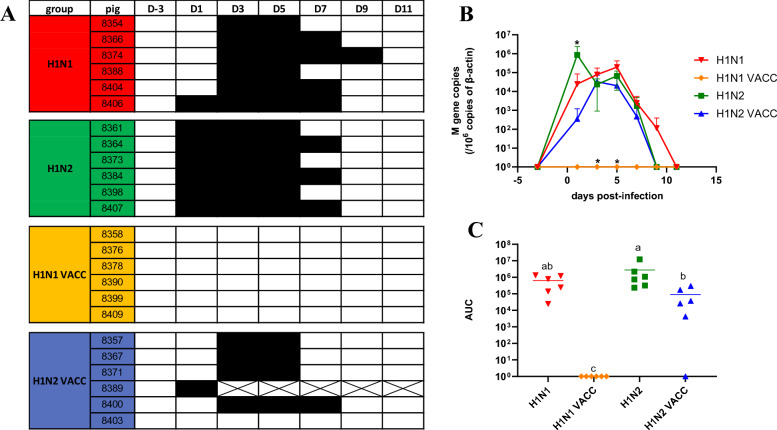
**Virus excretion in nasal secretions from D-3 to D11.** **A** Individual results of M-gene RT-qPCR on nasal swabs taken on infected pigs. Black squares indicate a swIAV positive sample and white squares indicate a swIAV negative sample. The crossed-out boxes indicate that the pig was dead. **B** Average of viral RNA amounts obtained in infected groups. * indicates that the group is significantly different from the other three. **C** Global amount of individual viral shedding. *AUC* area under the curve. The line indicates the mean AUC for the group. Significant differences between groups are indicated by different letters. For graphical representation purposes, value 1 has been assigned to negative samples. H1N1 and H1N1 VACC: groups challenged at D0 with the 272/20-H1_av_N1 strain (HA clade 1C.2.1). H1N2 and H1N2 VACC: groups challenged at D0 with the 154/20-H1_av_N2 strain (HA clade 1C.2.4).

After the first 272/20-H1_av_N1 or 154/20-H1_av_N2 challenge, all unvaccinated animals excreted swIAV but shedding occurred earlier in the H1N2 group than in the H1N1 group. At D1, the swIAV genome was detected in nasal swabs of all H1N2 pigs but only 1/6 H1N1 pigs (Figure 2A), resulting in a significantly higher amount of virus excreted in the H1N2 group than in the H1N1 group at that time (*p* = 0.03) (Figure 2B). At D3 and D5, swIAV was shed by all unvaccinated pigs, in similar amounts in both groups, and some pigs were still positive at D7 (in both groups) and at D9 (in the H1N1 group only). The global amounts of virus shedding (area under the curves) were similar in both unvaccinated groups (Figure 2C). Virus genome detections in individual oral fluids were similar to those evidenced from nasal swabs (Additional file 1). The viral genome was detected in rectal swabs taken at D3 in one H1N1 pig and two H1N2 pigs, but with high Cq values that did not allow quantification (data not shown).

No swIAV was detected in nasal swabs from H1N1 VACC pigs and only one oral fluid tested positive, at D4 (Figure 2 and Additional file 1). In contrary, virus shedding was not inhibited in the H1N2 VACC group, but only delayed as compared to the H1N2 group (Figure 2A). Whereas only one pig was positive at D1 in the vaccinated group against 6/6 in the unvaccinated one, swIAV was detected in 4/5 nasal swabs taken at D3 and D5, in similar genome copy numbers and with the same infectious titers than in unvaccinated pigs (10^4.4^ and 10^3.8^ TCID_50_/10^6^ copies of β-actin at D3, and 10^3.6^ and 10^4.1^ TCID_50_/10^6^ copies of β-actin at D5, respectively) (Figure 2A and B). The fifth pig was found positive at D4 in oral fluid (Additional file 1). Thus, the global amounts of H1N2 virus excreted in nasal swab supernatants (AUC) were found to be significantly lower in vaccinated group than in unvaccinated one (Figure 2C), but such a difference in virus shedding between both groups was not observed when measuring the virus genome in oral fluids (Additional file 1).

After the second challenge with the H1_av_N2 strain at D21, no virus shedding was detected in any group, whatever the primo-infection.

### Quantification of cytokines in sera

**Figure 3:**
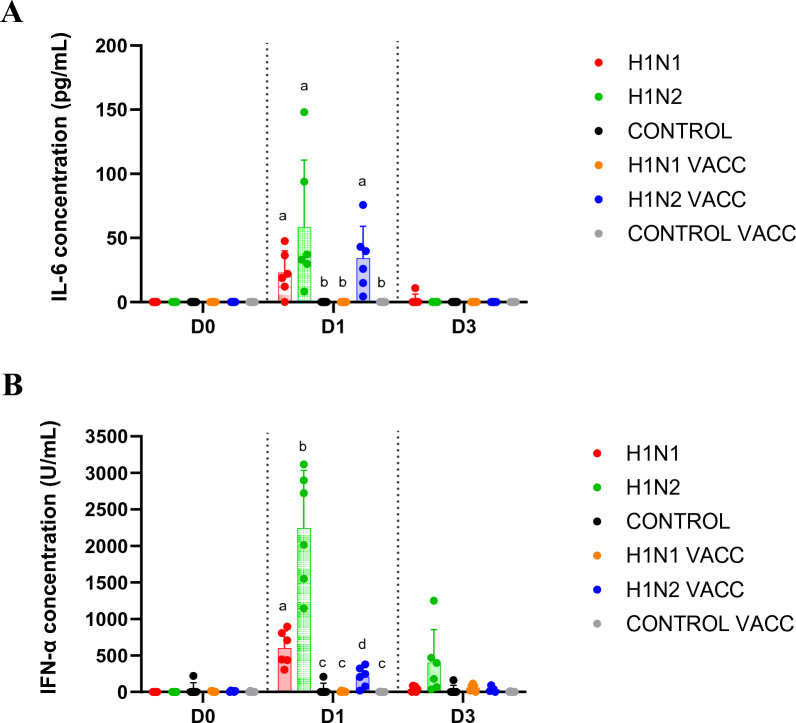
**Concentrations of cytokines in sera following the first swIAV infection.** Individual concentrations and mean ± standard deviation of concentrations of IL-6 (**A**) and IFN-α (**B**) in sera collected at D0, D1 and D3 in the six groups. The detection limits were 4.3 pg/mL for IL-6 and 25.1 U/mL for IFN-α. Significant differences between groups are indicated by different letters. H1N1 and H1N1 VACC: groups challenged at D0 with the 272/20-H1_av_N1 strain (HA clade 1C.2.1). H1N2 and H1N2 VACC: groups challenged at D0 with the 154/20-H1_av_N2 strain (HA clade 1C.2.4).

After the first swIAV challenge, IL-6 concentration significantly increased in sera taken at D1 in H1N1 and H1N2 groups, without statistical difference between both groups (*p* = 0.3) (Figure 3A). At the same day, IFN-α concentration was also higher in infected groups than in CONTROL group, but it was four times higher in the H1N2 group than in the H1N1 group, reaching 2241 ± 795 U/mL against 599 ± 237 U/mL, respectively (Figure 3B). At D3, IL-6 and IFN-α concentrations decreased or even retrieved the basal level measured in the CONTROL group.

In the H1N1 VACC group, productions of IL-6 and IFN-α after 272/20-H1_av_N1 infection were not evidenced in sera. On the contrary, peaks of both pro-inflammatory and anti-viral cytokines were clearly detected at D1 in the H1N2 VACC group. The IL-6 concentration was similar to that obtained in the unvaccinated groups, but the IFN-α concentration was reduced compared to the latter, reaching only 208 ± 138 U/mL.

### Evaluation of humoral immune responses in sera

**Figure 4:**
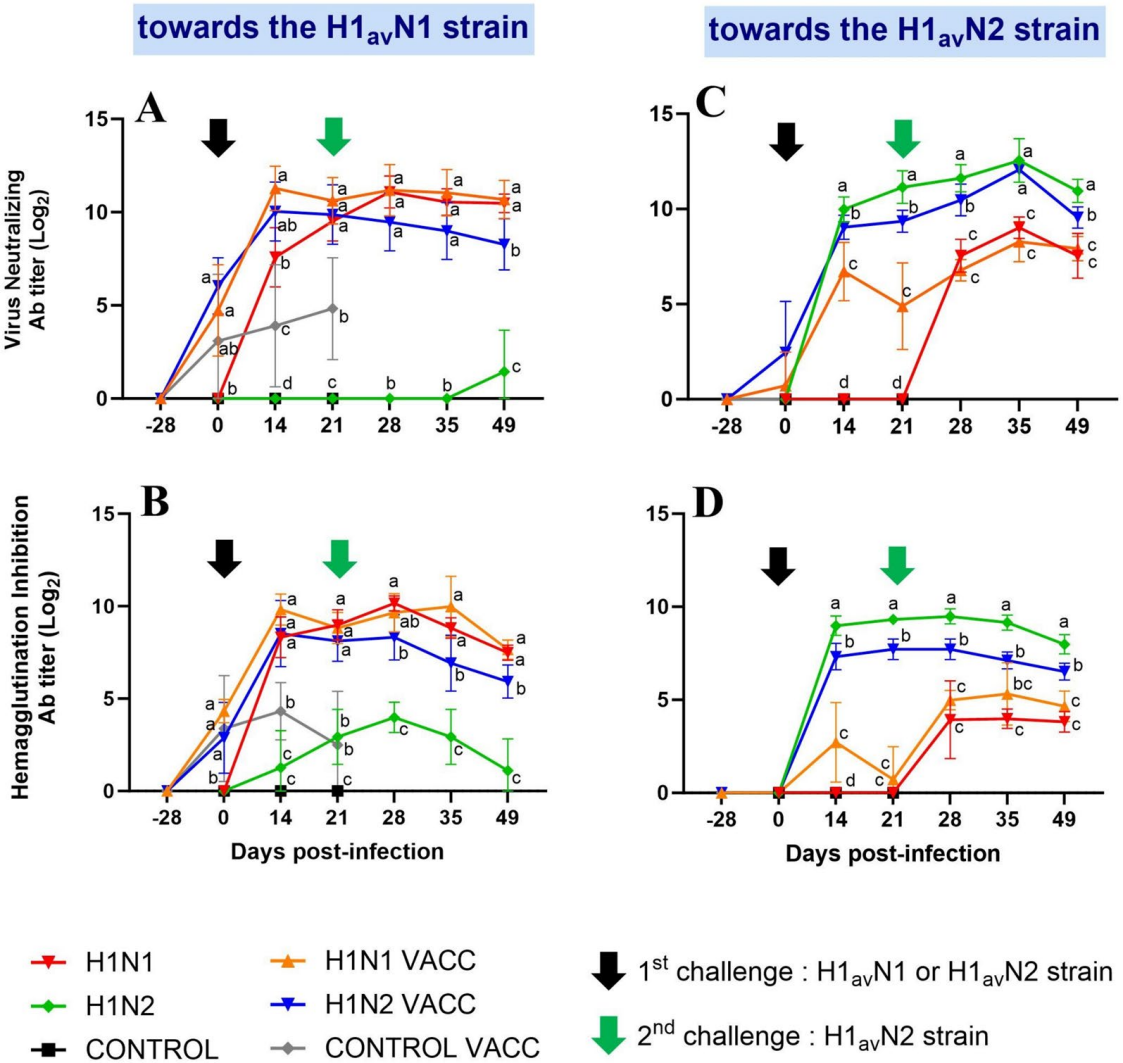
**Evolution of humoral immune responses over time.** Means (± standard deviation) of swIAV-specific antibodies (Ab) titers in the six groups, obtained by virus neutralization test (**A** and **C**) or by hemagglutination inhibition test (**B** and **D**), using either 272/20-H1_av_N1 (**A** and **B**) or 154/20-H1_av_N2 (**C** and **D**) as an antigen. The dates of the first 272/20-H1_av_N1 (HA clade 1C.2.1) or 154/20-H1_av_N2 (HA clade 1C.2.4) challenge and of the second 154/20-H1_av_N2 challenge are indicated by a black and a green arrow, respectively. Significant differences between groups are indicated by different letters. H1N1 and H1N1 VACC: groups challenged at D0 with the 272/20-H1_av_N1 strain (HA clade 1C.2.1). H1N2 and H1N2 VACC: groups challenged at D0 with the 154/20-H1_av_N2 strain (HA clade 1C.2.4).

Levels of 272/20-H1_av_N1-specific Ab were quantified in sera (Figures 4A, B), as well as levels of 154/20-H1_av_N2-specific Ab (Figures 4C, D).

No swIAV-specific Ab were detected in the CONTROL group throughout the study.

After the first H1_av_N1 or H1_av_N2 challenge, all unvaccinated animals presented Ab against the strain they were infected with at D14 and D21. At D21, VN and HI titers measured in homologous tests raised 9.52 ± 1.06 Log_2_ and 8.99 ± 0.82 Log_2_, respectively, in the H1N1 group (Figures 4A, B), and 11.14 ± 0.86 Log_2_ and 9.32 ± 0.00 Log_2_ in the H1N2 group (Figures 4C, D). The VN Ab levels measured after 154/20-H1_av_N2 infection were significantly higher than after 272/20-H1_av_N1 infection (*p* = 0.02), while the HI Ab levels were not statistically different. Very clearly, it appeared that post-infectious Ab directed against the 272/20-H1_av_N1 strain were unable to neutralize and cross-react with the 154/20-H1_av_N2 strain (Figures 4C, D). In the same way, antibodies produced post-H1_av_N2 infection were not able to neutralize the 272/20-H1_av_N1 strain but some cross-reactions were measured in HI tests with titers raising 2.93 ± 1.49 Log_2_ at D21 (Figures 4A, B).

Post-vaccination Ab titers measured at D0, i.e., one week after the immunization boost and before any challenge, were similar in the three vaccinated groups. The mean Ab titers measured in VN and HI tests using the 272/20-H1_av_N1 strain as an antigen were 4.62 ± 2.68 Log_2_ and 3.47 ± 2.08 Log_2_, respectively (Figures 4A, B). By contrast, post-vaccination Ab were not shown to neutralize or cross-react with the 154/20-H1_av_N2 strain (Figures 4C, D). After the first challenge, increases in 272/20-H1_av_N1-specific Ab titers were observed in the H1N1 VACC and H1N2 VACC groups, contrary to the CONTROL VACC group, for which the Ab levels remained stable until D21. Thus, titers measured in vaccinated and challenged groups were statistically different from the CONTROL VACC group at D14 and D21. At D21, the Ab levels able to neutralize or cross-react with the 272/20-H1_av_N1 antigen in these groups were similar to those obtained in the H1N1 group (Figures 4A, B). Titers in Ab able to neutralize or cross-react with the 154/20-H1_av_N2 antigen also increased after the challenge in the H1N2 VACC group. Thus, they reached a plateau from D14, with titers of 9.36 ± 0.58 Log_2_ and 7.72 ± 0.55 Log_2_ in VN and HI tests, respectively, to D21, that were significantly lower than those measured in the H1N2 group at the same time (Figures 4C, D). In H1N1 VACC group, an increase in the H1_av_N2-specific Ab titers was observed at D14. While lower than in both vaccinated and unvaccinated 154/20-H1_av_N2 infected groups, these titers were significantly higher than in H1N1, CONTROL VACC and CONTROL groups.

The second challenge with the 154/20-H1_av_N2 strain at D21 did not induce any changes in the H1_av_N1-specific humoral response in any group, as a significant decrease in HI Ab titers started to occur from D35 or D49 in the four groups equally (Figures 4A, B). Regarding the H1_av_N2-specific responses, VN Ab titers tended to increase slightly in the H1N2 and H1N2 VACC groups at D35 before to decrease, as revealed by lower titers measured at D49. However, these variations were not observed in HI tests. Finally, both H1N1 and H1N1 VACC groups acquired Ab able to neutralize and cross-react with the 154/20-H1_av_N2 antigen after the second challenge with 154/20-H1_av_N2 at D21. However, anti-H1_av_N2 Ab titers remained lower than those measured in the H1N2 and H1N2 VACC groups (Figures 4C, D).

### Evaluation of anti-NP antibody responses

**Figure 5:**
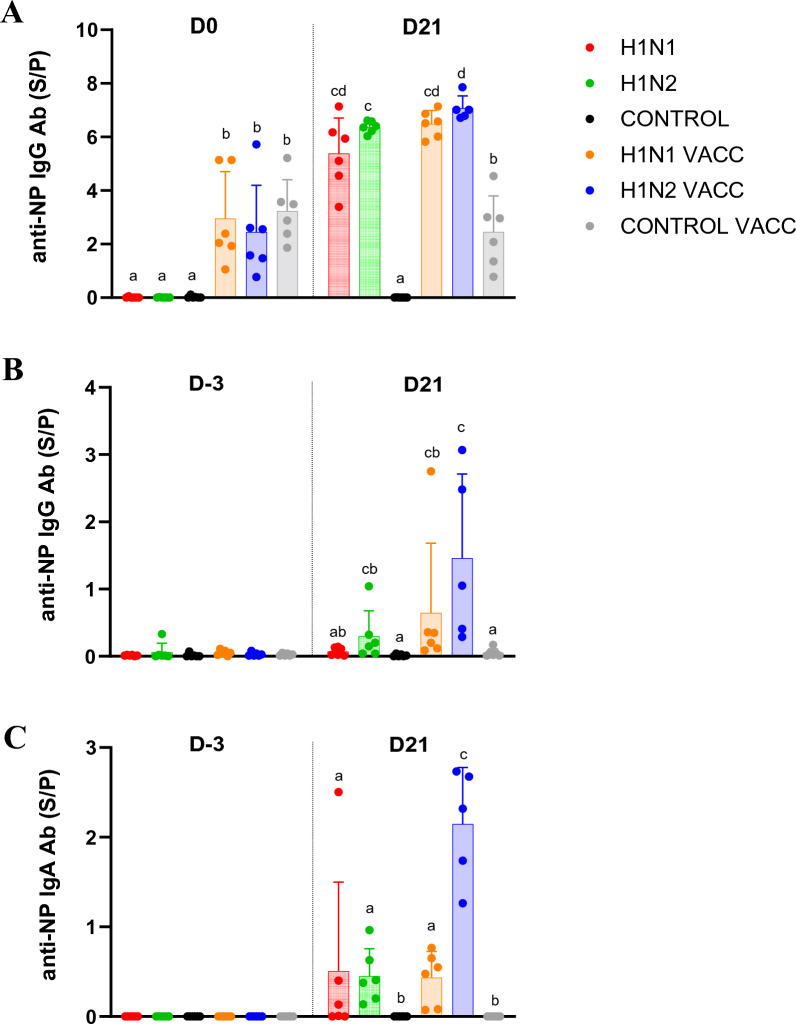
**ELISA detection of anti-NP antibodies in sera and/or nasal swabs taken before the first (at D-3 or D0) and the second (at D21) challenges.** Individual and mean (± standard deviation) ratio of sample to positive control (S/P) of (**A**) anti-NP IgG in sera, (**B**) anti-NP IgG in nasal swabs and (**C**) anti-NP IgA in nasal swabs. Significant differences between groups are indicated by different letters. H1N1 and H1N1 VACC: groups challenged at D0 with the 272/20-H1_av_N1 strain (HA clade 1C.2.1). H1N2 and H1N2 VACC: groups challenged at D0 with the 154/20-H1_av_N2 strain (HA clade 1C.2.4).

The levels of anti-NP antibodies present at the time of the first (D0) and second (D21) challenges either in sera or in nasal mucosa (nasal swabs) were assessed by ELISA (Figures 5A–C). The immunological status of pigs at the first challenge was assessed in sera taken at D0 but in nasal swabs taken at D-3, as no nasal swabs were taken at D0.

At the serological level, at D0, anti-NP IgG were detected in vaccinated animals, with the same levels in the three vaccinated groups, but not in unvaccinated groups (Figure 5A). Three weeks later, at D21, an increase in the anti-NP IgG response was observed in all infected animals whatever the challenge strain or the vaccination status. Thus, the four challenged groups showed similar S/P values at D21, which were significantly higher than the post-vaccine response measured at D0. In the CONTROL VACC group no change in the anti-NP IgG response was observed, since the mean S/P value was similar to that of D0 (*p* = 0.4).

Profiles of responses obtained in nasal swabs were quite similar for both IgG (Figure 5B) and IgA (Figure 5C). Contrary to detections in sera, anti-NP IgG or IgA were not measured in nasal swabs from vaccinated animals at D-3, as in unvaccinated animals. At D21, anti-NP responses were detected in the four infected groups, with higher S/P values for anti-NP IgA in the H1N2 VACC group as compared to the three other groups.

### Assessment of cellular immune responses

**Figure 6:**
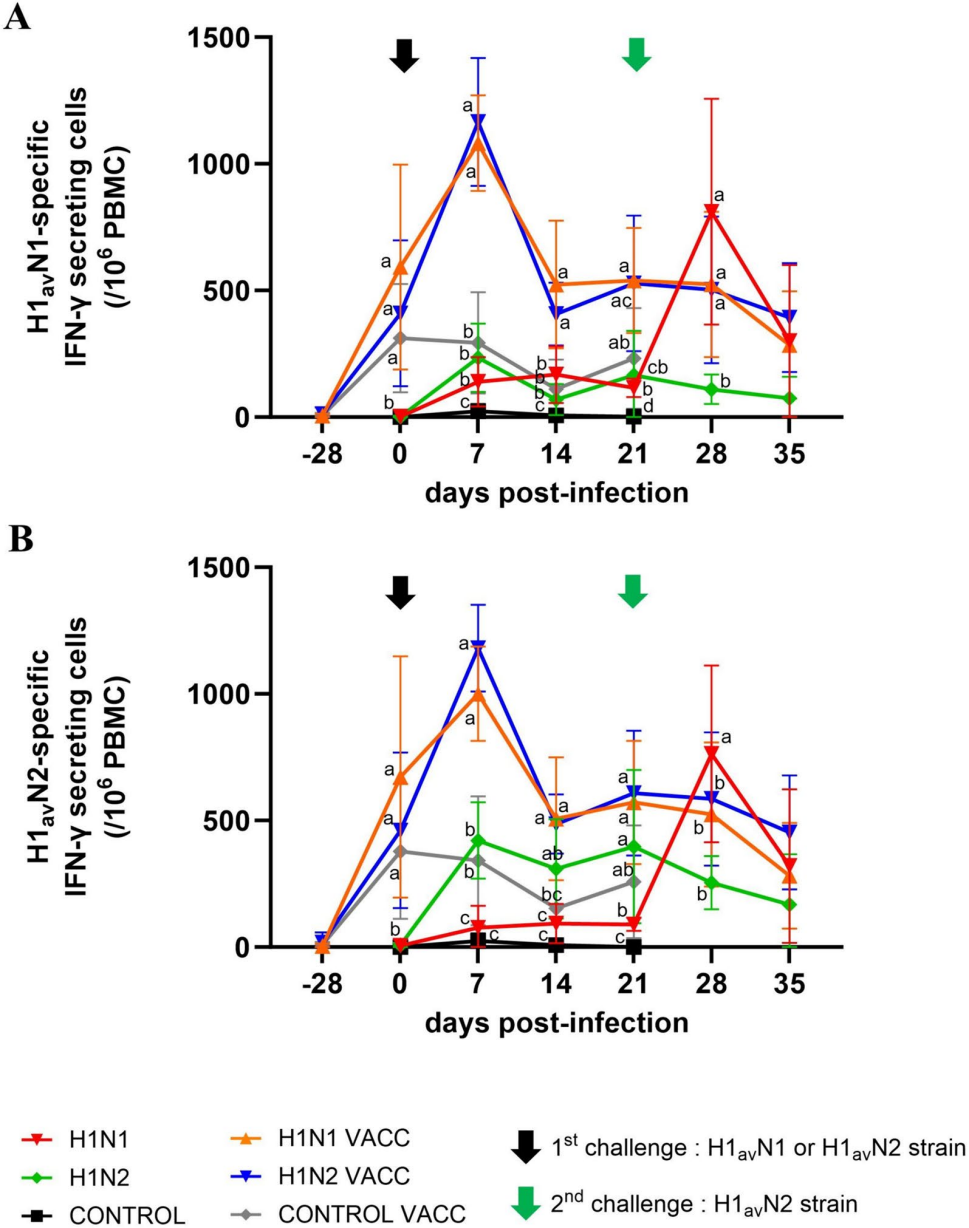
**Evolution of cellular immune responses over time.** Means (± standard deviation) of counts of IFN-γ secreting cells in the six groups, obtained after either 272/20-H1_av_N1 (**A**) or 154/20-H1_av_N2 (**B**) stimulation of peripheral blood mononuclear cells (PBMC). The dates of the first and second challenges are indicated by a black and a green arrow, respectively. Significant differences between groups are indicated by different letters. H1N1 and H1N1 VACC: groups challenged at D0 with the 272/20-H1_av_N1 strain (HA clade 1C.2.1). H1N2 and H1N2 VACC: groups challenged at D0 with the 154/20-H1_av_N2 strain (HA clade 1C.2.4).

The swIAV-specific cellular responses were followed by IFN-γ ELISPOT, after PBMC stimulation with the 272/20-H1_av_N1 strain (Figure 6A) or the 154/20-H1_av_N2 strain (Figure 6B).

No swIAV-specific cellular response was detected in the CONTROL group throughout the study. The average number of IFN-γ SC (IFN-γ secreting cells / 10^6^ PBMC) remained very low, close to the background level, comprised between 0 ± 2 and 24 ± 31 depending on the sampling day, whatever the virus used as a stimulating antigen.

In both the unvaccinated H1N1 and H1N2 groups, a cellular response specific to the first swIAV challenge was established from D7. The numbers of IFN-γ SC/10^6^ PBMC measured in homologous test at D7 were 140 ± 97 in the H1N1 group (Figure 6A) and 421 ± 151 in the H1N2 group (Figure 6B). These responses remained quite stable until D21 and significantly higher in the H1N2 group as compared to the H1N1 group at D21. IFN-γ SC were also numbered in both infected groups in heterologous conditions. At D7, 77 ± 86 IFN-γ SC were counted in the H1N1 group after PBMC stimulation with the 154/20-H1_av_N2 strain (Figure 6B) and 234 ± 135 IFN-γ SC were counted in the H1N2 group after PBMC stimulation with 272/20-H1_av_N1 (Figure 6A). Numbers of IFN-γ SC were lower in heterologous conditions than in homologous conditions, while not statistically different whatever the date.

In the three vaccinated groups, a similar cell-mediated immune response was evidenced following vaccination whatever the strain used for PBMC stimulation (Figures 6A, B). Thus, at D0, mean numbers of IFN-γ SC/10^6^ PBMC were 438 ± 317 and 504 ± 362 after stimulation with 272/20-H1_av_N1 strain or 154/20-H1_av_N2 strain, respectively. After the challenge, a boost effect was similarly observed in the two vaccinated/challenged groups at D7, whatever the stimulating virus used in the ELISPOT. Numbers of 272/20-H1_av_N1-specific IFN-γ SC reached 1083 ± 189 and 1165 ± 252 in H1N1 VACC and H1N2 VACC, respectively, while numbers of 154/20-H1_av_N2-specific IFN-γ SC reached 1001 ± 186 and 1181 ± 171 in these groups, respectively. At D14, all numbers decreased to pre-challenge levels.

After the second challenge with 154/20-H1_av_N2 at D21, no boost effect was observed in any group, except the H1N1 group, for which peaks of H1_av_N1-specific and H1_av_N2-specific IFN-γ SC numbers were observed at D28, reaching 811 ± 446 and 763 ± 349 IFN-γ SC, respectively. Then, at D35, the cellular response decreased in all groups.

## Discussion

This study aimed to characterize antigenic and pathogenic properties of the new H1_av_N2 genotype that emerged in France in 2020, in order to provide new knowledge that could contribute to explain why this virus propagated so rapidly and efficiently in the pig population. As observed in cross-HI tests, the 154/20-H1_av_N2—HA-1C.2.4 strain used as a representative challenge strain in this study presented a marked antigenic distance from other HA-1C viruses previously detected in France, which mainly belonged to HA clade 1C.2.1. The new genotype was also distant from previous H1_av_N2—HA 1C.2.4 strains sporadically detected in 2015, as well as from the Respiporc^®^ Flu3 H1_av_ antigen that belongs to HA clade 1C.2.2. As no non-specific reaction induced by the N2 was observed, we can assume that these observations were due to HA and not NA. These results were consistent with data accumulated since 2020 by the National Reference Laboratory, which suspected that this new genotype was antigenically distant from other H1_av_ viruses isolated in France before, and reported a limited cross-reactivity in HI tests with antisera obtained from vaccinated animals [10].

This antigenic distance between H1_av_N2-2020 and other H1_av_ viruses was here confirmed in cross-VN tests, raising the question of a potential escape from pre-existing immunity and/or vaccine protection. It was with the aim of answering this question that we conducted the in vivo study presented here.

In unvaccinated pigs, the 154/20-H1_av_N2 strain induced an acute respiratory infection characterized by marked clinical signs, inflammatory and antiviral responses. Compared to the 272/20-H1_av_N1 infection, the 154/20-H1_av_N2 infection was noticeably more severe, with animals showing more coughing and sneezing, over a longer period of time. In the same way, a higher production of IFN-α was observed, which is consistent with the severity of clinical signs, as already described [26]. These observations in experimental conditions were also consistent with reports obtained from the field during the epidemic phase in 2020, thanks to the national surveillance network Résavip. Indeed, veterinarians considered the intensity of the influenza illness was severe (versus normal) in 55.6% of herds affected by the H1_av_N2-HA 1C.2.4 virus, while in only 25% of herds when infected with other swIAVs [10]. Shedding of the 154/20-H1_av_N2 virus began as early as one day after the inoculation and the virus was not detected any longer in individual nasal swabs than the 272/20-H1_av_N1 virus. Multiplication seemed limited to the respiratory tract; no digestive tropism was demonstrated via rectal swab analyses.

Regarding the adaptive immune response, the kinetic and intensity of the humoral responses after the 154/20-H1_av_N2 infection were similar to those induced after the 272/20-H1_av_N1 infection, at mucosal and serological compartments. Similar levels of IgA and/or IgG were measured in nasal swabs and in sera, however the anti-HA antibodies which are those mainly detected by HI and VN tests, were unable to cross-react thus confirming the important antigenic distance between both strains. Inversely, the 154/20-H1_av_N2 infection induced a cell-mediated immune response able to cross–react with the 272/20-H1_av_N1 strain, and vice-versa. These results are consistent with the fact that, contrary to the humoral immune response which mainly target epitopes present on the HA surface protein, the immunodominant epitopes recognized by the cellular response are located in the internal proteins—M1, NP, PA and PB1—which are relatively conserved between swIAV of same viral lineage [27, 28]. Thus, the two strains of our study showed 94.27–100% of homology for these proteins, therefore explaining the cross-reactions of T cells.

At D21, all pigs were challenged with the 154/20-H1_av_N2 strain in order to evaluate the protection conferred by a heterologous (272/20-H1_av_N1) or a homologous (154/20-H1_av_N2) primo-infection. Despite the absence of anti-H1_av_N2 neutralizing antibodies in H1N1 pigs after the first challenge, all these animals seemed protected from the subsequent H1_av_N2 infection since no clinical signs and no virus shedding were observed. These results suggested that the cell-mediated immune response induced by the 272/20-H1_av_N1 infection, in combination with non-neutralizing antibodies directed against internal proteins, as anti-NP antibodies detected in nasal mucosa and in sera, were sufficient to provide a protection against the 154/20-H1_av_N2 challenge that was given three weeks after the primo-infection. H1N1 pigs developed an anti-H1_av_N2 adaptive immune response after the heterologous infection at D21, with no boost of the anti-H1_av_N1 adaptive response. Thus, we did not evidence here the “original antigenic sin” that can sometimes occur in case of successive heterologous infections [29]. In H1N2 pigs, we also did not observed any boost effect on anti-H1_av_N2 adaptive immune responses after the second (homologous) challenge. This was in accordance with previous studies investigating successive homologous H1_av_N1 infections [30, 31] and suggested that the systemic immune system was no more stimulated by viral antigen during re-challenge with homologous strains. It can be noted that none of the H1N1 pigs presented any lung lesions at the end of the study, confirming they counteracted very well the 272/20-H1_av_N1 infection and were not infected by the 154/20-H1_av_N2 inoculated in a second time. By contrast, pigs from the H1N2 group exhibited lung lesions at D25. It is more likely that these macroscopic lesions were residual lesions from the severe pneumonia that affected the animals after the first 154/20-H1_av_N2 infection, rather than lesions caused by the second homologous inoculation. Indeed, these H1N2 animals were totally protected from a second infection, since no clinical signs or viral excretion was highlighted from D21, suggesting that the immune responses induced by the first infection, i.e. memory T cells and specific antibodies, quickly neutralized the virus before it was able to infect cells productively.

Given that 154/20-H1_av_N2 was more distant genetically and antigenically from the Respiporc^®^ Flu3 vaccine HA-1C antigen than 272/20-H1_av_N1, it could be hypothesized that vaccination was less efficient to protect pigs from infection with the new H1_av_N2 genotype than with the H1_av_N1 virus. The in vivo study demonstrated that all vaccinated animals were severely affected by the 154/20-H1_av_N2 but not the 272/20-H1_av_N1 infection. Even if coughs and sneezes were less frequent the day after 154/20-H1_av_N2 inoculation in vaccinated pigs compared to unvaccinated ones, respiratory disorders were observed for the same period of time in both groups. The vaccination also had no beneficial effect on the growth retardation due to the 154/20-H1_av_N2 infection. Moreover, the necropsy carried out at D1 on the pig euthanized for ethical issue showed an important pneumonia reaching lower lobes of the lung, which was in line with the detection of a peak of pro-inflammatory IL-6 cytokine in H1N2 VACC animals at D1. Thus, in our experimental conditions, the 154/20-H1_av_N2 virus multiplied into the vaccinated hosts, was excreted for a week and induced a huge lung inflammation accompanied of severe clinical signs. All these observations indicated that the vaccination with the trivalent inactivated vaccine failed to protect animals from the 154/20-H1_av_N2 infection outcomes, contrary to what was observed in the case of the 272/20-H1_av_N1 infection. However, at the end of the study, the H1N2 VACC pigs no longer showed any pneumonia lesion, unlike unvaccinated animals, suggesting that vaccination could help animals to recover more quickly.

Although the 154/20-H1_av_N2 strain showed antigenic distance from both 272/20-H1_av_N1 virus and vaccine antigen, we observed that, in our experimental conditions, 272/20-H1_av_N1 pre-infected animals were protected from a subsequent infection with the 154/20-H1_av_N2 strain, whereas vaccinated animals were not. Both H1N1 and H1N2 VACC groups presented anti-HA antibodies unable to cross-react with the 154/20-H1_av_N2 virus but a cross-reacting cellular immune response. However, comparing other parameters of pre-existing immunity at the time of 154/20-H1_av_N2 challenge, i.e. at D0 for H1N2 VACC animals and at D21 for H1N1 animals, it could be hypothesized that at least two important factors would have contributed in leading to different levels of protection. Firstly, the H1N1 animals showed a much greater quantity of non-neutralizing antibodies, such as anti-NP IgG, in sera. Non-neutralizing antibodies can contribute to antibody-mediated effector mechanisms, such as antibody-dependent cellular cytotoxicity or antibody-dependent cellular phagocytosis, through the interaction of their Fc region with other components of the immune system such as NK cells, macrophages or neutrophils, resulting in their activation [32, 33]. Thus, it has been shown that influenza virus-specific CD8 + T cells combined with non-neutralizing antibodies elicited heterosubtypic protective immunity in mice [34]. It could be supposed that, in our study, the quantity of non-neutralizing antibodies in the H1N2 VACC animals was insufficient to activate this cooperative protection while this happened after 272/20-H1_av_N1 infection. Secondly, contrary to 272/20-H1_av_N1 infection, vaccination with an inactivated vaccine did not induce any mucosal immunity [17]. IgA antibodies contribute to mucosal immunity notably by preventing the entry of the virus into the epithelial cells [35] and, interestingly, it has been shown that they were more cross-reactive than the IgG [36]. It could thus be supposed that IgA antibodies present in the respiratory mucosae of H1N1 pigs participated to cross-protection against the 154/20-H1_av_N2 infection.

In conclusion, our findings demonstrated that the new H1_av_N2—HA 1C.2.4 genotype was more virulent than the H1_av_N1—HA 1C.2.1 previously predominant in the pig population in France. Further analyses are now necessary to investigate the virus-dependent mechanisms responsible for these severe clinical manifestations and understand the differences observed with the H1_av_N1 outcomes. In our experimental conditions, the pre-existing immunity resulting from a previous swIAV-infection protected the animals against a H1_av_N2—HA 1C.2.4 infection 3 weeks later, but additional investigations would be required to determine the duration of this protection. Furthermore, maternal derived antibodies have been shown to interfere with the establishment of the humoral immune response following a swIAV infection in piglets [31]. It would thus be interesting to study the impact of maternally derived antibodies in the cross-protection observed here. Our study showed that the inactivated trivalent vaccine did not protect the animals against H1_av_N2—HA 1C.2.4 infection in our controlled conditions, and that vaccinated animals excreted as much virus as unvaccinated ones. The escape of the vaccine protection could explain in part the rapid spread of the H1_av_N2 – HA 1C.2.4 viruses in the pig population. As such an escape from pre-existing immunity was not reported in other European countries where virus strains with HA clade 1C.2.4 have been also detected, once may ask if the specific pattern of mutations observed in French strains would have led to this antigenic drift among 1C.2 lineage. Further investigations would be necessary to study cross-reactions between 1C.2.4 strains from different countries, to verify this hypothesis. In any case, in Europe, the strains that are included in swine influenza vaccines are rarely updated [37], while our results clearly raise serious concerns about the necessity to update the vaccines antigens to match the strains in circulation, or to change the vaccine strategy to improve cross-protection. However, the best solution to avoid a new epizootic and the circulation of a new enzootic virus would be to prevent the introduction of a new genotype into the territory by reinforcing quarantine measures and swIAV infection monitoring of live swine traded between European countries for reproduction purposes.

### Supplementary Information

**Additional file 1. Detection of swIAV genome in individual oral fluids from D-3 to D11. (A)** Individual results of M-gene RT-qPCR on oral fluids taken on infected pigs. Black squares indicate the detection of swIAV genome and white squares indicate that the virus genome was not detected. The crossed-out boxes indicate that the pig was dead. (**B**) Average of viral RNA amounts obtained in infected groups. (**C**) Global amount of individual viral shedding. AUC = area under the curves. The line indicates the mean AUC for the group. Significant differences between groups are indicated by different letters. For graphical representation purposes, value 1 has been assigned to negative samples. H1N1 and H1N1 VACC: groups challenged at D0 with the 272/20-H1_av_N1 strain (HA clade 1C.2.1). H1N2 and H1N2 VACC: groups challenged at D0 with the 154/20-H1_av_N2 strain (HA clade 1C.2.4).

## Data Availability

The datasets used and/or analysed during the current study are available from the corresponding author on reasonable request.
