# Supplementary figures and images for: Pathogenicity and escape to pre-existing immunity of a new genotype of swine influenza H1N2 virus that emerged in France in 2020
Source: Vet Res. 2024 May 21;55:65. doi: 10.1186/s13567-024-01319-5 (PMC11110284; doi:10.1186/s13567-024-01319-5)

A

| group     | pig  | D-3 | D1 | D2 | D3 | D4 | D7 | D8 | D9 | D10 |
|-----------|------|-----|----|----|----|----|----|----|----|-----|
| H1N1      | 8354 |     |    |    |    |    |    |    |    |     |
|           | 8366 |     |    |    |    |    |    |    |    |     |
|           | 8374 |     |    |    |    |    |    |    |    |     |
|           | 8388 |     |    |    |    |    |    |    |    |     |
|           | 8404 |     |    |    |    |    |    |    |    |     |
|           | 8406 |     |    |    |    |    |    |    |    |     |
| H1N2      | 8361 |     |    |    |    |    |    |    |    |     |
|           | 8364 |     |    |    |    |    |    |    |    |     |
|           | 8373 |     |    |    |    |    |    |    |    |     |
|           | 8384 |     |    |    |    |    |    |    |    |     |
|           | 8398 |     |    |    |    |    |    |    |    |     |
|           | 8407 |     |    |    |    |    |    |    |    |     |
| H1N1 VACC | 8358 |     |    |    |    |    |    |    |    |     |
|           | 8376 |     |    |    |    |    |    |    |    |     |
|           | 8378 |     |    |    |    |    |    |    |    |     |
|           | 8390 |     |    |    |    |    |    |    |    |     |
|           | 8399 |     |    |    |    |    |    |    |    |     |
|           | 8409 |     |    |    |    |    |    |    |    |     |
| H1N2 VACC | 8357 |     |    |    |    |    |    |    |    |     |
|           | 8367 |     |    |    |    |    |    |    |    |     |
|           | 8371 |     |    |    |    |    |    |    |    |     |
|           | 8389 |     |    |    |    |    |    |    |    |     |
|           | 8400 |     |    |    |    |    |    |    |    |     |
|           | 8403 |     |    |    |    |    |    |    |    |     |

B

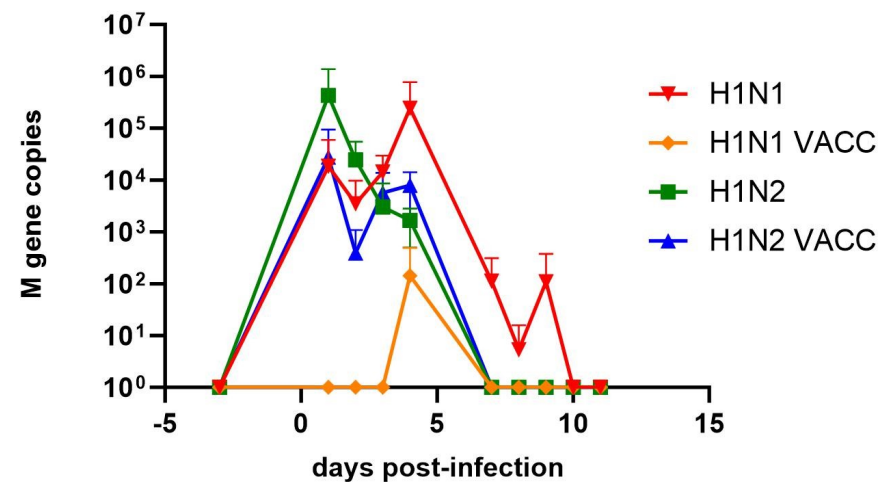

C

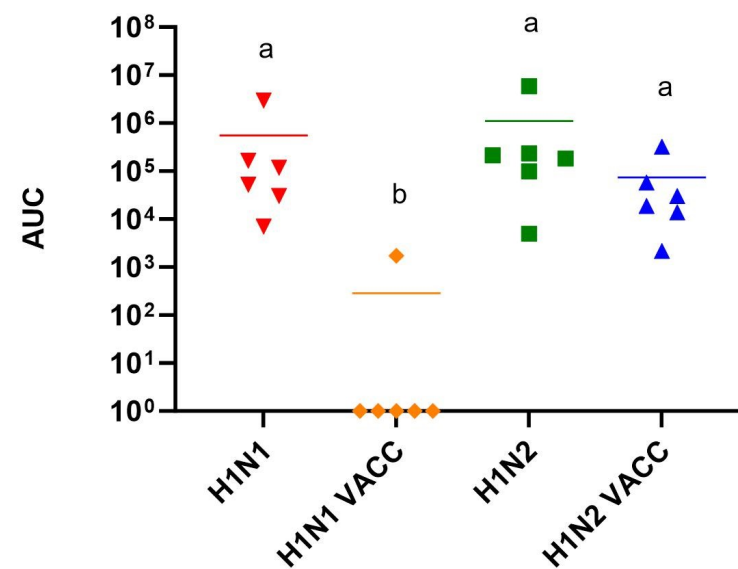

Supplement: Supplementary file 1 — Additional file 1. Detection of swIAV genome in individual oral fluids from D-3 to D11. (A) Individual results of M-gene RT-qPCR on oral fluids taken on infected pigs. Black squares indicate the detection of swIAV genome and white squares indicate that the virus genome was not detected. The crossed-out boxes indicate that the pig was dead. (B) Average of viral RNA amounts obtained in infected groups. (C) Global amount of individual viral shedding. AUC = area under the curves. The line indicates the mean AUC for the group. Significant differences between groups are indicated by different letters. For graphical representation purposes, value 1 has been assigned to negative samples. H1N1 and H1N1 VACC: groups challenged at D0 with the 272/20-H1avN1 strain (HA clade 1C.2.1). H1N2 and H1N2 VACC: groups challenged at D0 with the 154/20-H1avN2 strain (HA clade 1C.2.4). [file 13567_2024_1319_MOESM1_ESM.pdf]
